# Sea lice as a density-dependent constraint to salmonid farming

**DOI:** 10.1098/rspb.2012.0084

**Published:** 2012-02-08

**Authors:** Peder A. Jansen, Anja B. Kristoffersen, Hildegunn Viljugrein, Daniel Jimenez, Magne Aldrin, Audun Stien

**Affiliations:** 1 Norwegian Veterinary Institute, PO Box 750 Sentrum, 0106 Oslo, Norway; 2 Norwegian Computing Center, PO Box 114 Blindern, 0314 Oslo, Norway; 3 Norwegian Institute for Nature Research, Fram—High North Research Centre for Climate and the Environment, 9295 Tromsø, Norway; 4 Department of Informatics, University of Oslo, PO Box 1080 Blindern, 0316 Oslo, Norway; 5 Centre for Ecological and Evolutionary Synthesis (CEES), Department of Biology, University of Oslo, PO Box 1066 Blindern, 0316 Oslo, Norway; 6 Department of Mathematics, University of Oslo, PO Box 1053 Blindern, 0316 Oslo, Norway

**Keywords:** *Lepeophtheirus salmonis*, host density, parasite transmission, population dynamics

## Abstract

Fisheries catches worldwide have shown no increase over the last two decades, while aquaculture has been booming. To cover the demand for fish in the growing human population, continued high growth rates in aquaculture are needed. A potential constraint to such growth is infectious diseases, as disease transmission rates are expected to increase with increasing densities of farmed fish. Using an extensive dataset from all farms growing salmonids along the Norwegian coast, we document that densities of farmed salmonids surrounding individual farms have a strong effect on farm levels of parasitic sea lice and efforts to control sea lice infections. Furthermore, increased intervention efforts have been unsuccessful in controlling elevated infection levels in high salmonid density areas in 2009–2010. Our results emphasize host density effects of farmed salmonids on the population dynamics of sea lice and suggest that parasitic sea lice represent a potent negative feedback mechanism that may limit sustainable spatial densities of farmed salmonids.

## 1. Introduction

Global fisheries catches have been relatively stable over the last two decades [1]. Depletion of many fish stocks [2–5] and estimates of the natural primary production in the oceans [6] suggest that there is little prospect for growth in fisheries catches in the near future. Over the same period, production volume in aquaculture has grown at a rate far exceeding that of the global human population [7], suggesting that aquaculture has the potential to supply animal proteins in response to the growing demands [7–9]. It is recognized that current intense fish farming practices can cause pollution and disease problems, escaped fish have negative impacts on wild stocks, and that farming of carnivorous species puts pressure on wild fish populations used for feed [3,8,10,11]. Together with space limitations, these factors have been predicted to set natural limits to sustainable intensities of fish farming.

The principle of density-dependent disease transmission rates is a cornerstone in the epidemiological theory of infectious diseases [12]. It is supported by empirical studies in human, agricultural and wildlife systems [12–15] and on viral, bacterial and macroparasitic infectious diseases [12]. The expectation that disease problems in aquaculture will increase as the density of farmed fish increases is therefore well founded. However, while examples of infectious disease problems in aquaculture are plenty [16], there is a lack of studies evaluating the importance of host densities for disease transmission in full-scale production systems [17–19]. Thus, there is little empirical evidence in support of the notion that diseases may become a main factor limiting future growth in the aquaculture industry.

**Figure 1.**
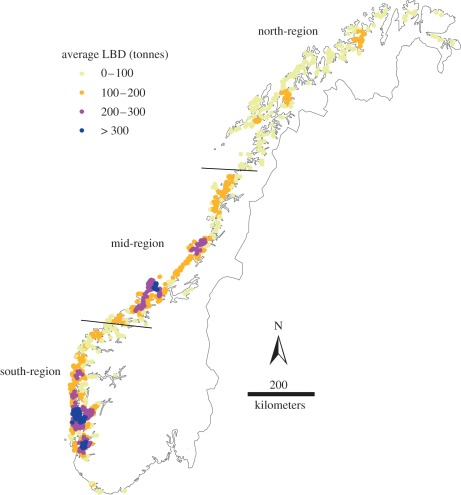
Average local biomass density (LBD) of farmed salmonids surrounding each salmon farm included in the study. For each farm location and month, the Gaussian kernel density of farmed salmonids (tonnes) within a seaway distance of 40 km was estimated using the density() function in R, with a standard deviation of 20.25 km (and truncated at 40 km). The mean over all months is shown in the map for each farm. Average LBD (tonnes): light yellow region, 0–100; dark yellow region, 100–200; pink region, 200–300; dark blue region, greater than 300.

Marine salmon farming in Norway is one of the most industrialized fish farming enterprises in the world [20], producing close to one million tonnes of Atlantic salmon (*Salmo salar*; 0.93 million tonnes) and rainbow trout (*Oncorhyncus mykiss*; 0.05 million tonnes) in 2010 [21]. Standing stock biomass of farmed salmon has roughly doubled over the period 2002–2010 (see electronic supplementary material, figure S1) and farmed salmon were recently estimated to outnumber return migrating wild salmon by a factor of 250–700 in Norwegian coastal waters [16]. Still, the spatial density of farmed salmon varies substantially along the coast with generally lower densities in the north than in the southwest (figure 1). Growing concern is raised about the sustainability of salmon farming on this large scale, in particular with regard to transmission of parasitic sea lice [22–24].

Farmed salmonids are grown in floating net pens allowing free water exchange, and hence pathogen exchange, with the environment. Sea lice are naturally occurring ectoparasitic copepods that transmit directly between hosts by planktonic larvae. They have temperature-sensitive development and reproductive rates [25], and they occur frequently in marine salmonid farms. Sea lice infections on salmonids in Norway are dominated by the salmonid specialist *Lepeophtheirus salmonis*, while the generalist *Caligus elongatus* occur at lower abundances [26,27]. The potential negative impacts of sea lice of farm origin on wild salmon populations cause environmental concerns and conflicts with wild salmon stakeholders [28,29]. Empirical studies support the hypothesis that sea lice of farm origin is a main source of infection in wild salmon [24,27,30]. Less focus has been on transmission between salmon farms. One reason for this may be that sea lice control, e.g. using efficient chemotherapeutic treatments or cleaner fish of the family *Labridae*, has been successful at keeping sea lice abundances at sub-clinical levels in salmon farms. However, reports on the development of chemotherapeutic resistance in sea lice populations [31,32] suggest that this situation may change.

Here, we report from a first large-scale study of the effect of farmed fish densities on parasite abundances and control efforts in a highly industrialized fish farming system. In our study system, the density of infective sea lice larvae in the waters around the fish farms is likely to be the main determinant of the infection rate (i.e. the force of infection [12]) experienced by individual hosts. We reason that a large population of hosts in surrounding waters is likely to harbour a large population of adult parasites and thereby produce more infective larvae than a smaller population of hosts. In addition, infective larvae densities are likely to be affected by temperature since both fecundity and generation time of sea lice are temperature dependent [25]. Hence, our expectations were that sea lice infection rates should be high in fish farms surrounded by a high density of farmed salmonids and/or in warm waters, and that sea lice infection rates should be lower in areas with low salmonid densities or colder waters. We also expected a strong seasonal pattern in sea lice infection rates, driven by seasonal fluctuations in water temperature. Our data do not contain direct measures of infection rates, but estimates of sea lice abundances and control intervention efforts. We analyse sea lice abundance as a proxy for infection rate. The rationale behind this is that the rate of change in sea lice abundance over a given period of time will be determined by infection rate, given a constant sea lice mortality rate. Hence, when controlling for abundance backwards in time as well as changes in sea lice mortality, different levels of abundance will reflect different levels of infection rate. We assume that major changes in sea lice mortality arise from control interventions undertaken to accommodate regulations on the maximum legal thresholds of sea lice abundance (see electronic supplementary material, methods), which therefore needs to be taken into account when interpreting infection rate based on sea lice abundance data.

We analyse monthly data on sea lice counts, parasite control efforts and production volume from all active marine salmonid farms along the coast of Norway in the years 2002–2010. We first investigate the importance of local biomass density (LBD) of farmed salmonids on both the average abundances of infection and on control intervention efforts in an analysis of data aggregated at a regional spatial scale and annual temporal scale. Thereafter, we investigate the effect of LBD, control interventions and temperature on the temporal variability of abundance of infection in individual farms. We use autoregressive models to capture the temporal autocorrelation in lice abundances within farm sites. Both Atlantic salmon and rainbow trout are parasitized by sea lice, but rainbow trout tends to have lower infection levels [33]. In addition, sea lice abundance tends to increase with fish size owing to an increasing period of exposure to infection with age and/or owing to changes in infection rates with size [34]. We, therefore, included farmed species of salmonids and mean fish weights in the analyses.

## 2. Methods

### (a) Data

Atlantic salmon (*Salmo salar*) and rainbow trout (*Oncorhynchus mykiss*) are farmed on a large scale in Norway [16]. For simplicity, we term this salmonid farming in this paper. Operators of salmonid farms must have a legal concession authorized by the Directorate of Fisheries (DFF; www.fiskeridir.no) and all farms are registered with a geo-reference in the aquaculture licence register, which is available at DFF's website. For farms that actively farm salmonids in the marine environment, it is mandatory to report key statistics on their fish stocks, fish health-related statistics and water temperature at a depth of 3 m, to responsible authorities on a monthly basis. The present data cover monthly reports from all farmed stocks of salmonids in marine waters in Norway over the period January 2002 to December 2010.

Sea lice infections may be by *Lepeophtheirus salmonis* or *Caligus elongatus* [26,27], hence we use the term ‘sea lice’ in this paper. Sea lice infections in farmed salmonids are regulated through maximum thresholds to abundance of mobile stages of lice (see electronic supplementary material, methods). To enforce these regulations, farmers are instructed to count sea lice on farmed salmonids at regular intervals and report the highest mean count during a month. The mean count of sea lice from a sample of 20 fish from one net pen (before August 2009) or the mean of means from samples of 10 fish from multiple net pens (from August 2009) was reported (see electronic supplementary material, methods). To get an integer number to be used in the present statistical count model, the dependent variable was derived by multiplying reported mean counts of sea lice by 20 and rounding off this to the nearest integer (see electronic supplementary material, methods).

The total dataset consists of 61161 reported mean counts of mobile sea lice from a total of 1442 salmon farms (electronic supplementary material, figure S2). Monthly mean counts of sea lice were highly aggregated and for each month between 12.4 and 51 per cent (34.3% for the total dataset) of the active farms reported zero sea lice.

Monthly statistics reported by salmonid farms and explored directly as explanatory variables for the sea lice counts included: mean fish weight; water temperatures; whether farmed species was Atlantic salmon or rainbow trout; whether medical sea lice treatment was applied; or whether cleaner fish of the family *Labridae* were applied. In addition, we estimated a proxy variable for farm site salinity, expressing the relative exposure to freshwater for given farm sites. This latter variable was only used to analyse a subset of the data comprising 50 per cent of the farm sites with the lowest estimates for freshwater exposure (see electronic supplementary material, methods and table S4).

The reported number of fish and mean fish weight in the farm stocks were used to calculate farm-specific LBD of farmed salmonids. For each farm in each month, stock biomass was calculated as the number of fish multiplied by mean weight. The LBD surrounding each farm in each month was calculated as a kernel density of stock biomasses within 40 km seaway distance of given farms, where the biomass on the farm for which LBD was estimated was not included. A Gaussian kernel density function (density() in the statistical package R [35]) with a standard deviation of 20.25 km, and which was truncated at 40 km, was used for the LBD calculations. Pair wise seaway distances between salmon farms were compiled from Kristoffersen *et al.* [36]. We did not distinguish between farmed species of salmonids in the LBD estimations.

Further details on data compilation and processing are given in the electronic supplementary material.

### (b) Exploratory analyses of region-level data

To explore the data on an aggregated level, the dataset was subdivided into three geographical regions (figure 1); the north-region (all farms north of 67° latitude), the mid-region (farms between latitudes 67°–62° 35 min) and the south-region (all farms south of 62° 35 min latitude). Further subdivisions of the data were done on a monthly basis (figure 4; electronic supplementary material, figure S4), or a yearly mean basis (figure 3), for farms located in areas with low (less than 33.3 percentile), medium (33.3–66.6 percentiles) and high (greater than 66.6 percentile) LBD. We analysed dependencies of sea lice counts, medical treatments and the use of cleaner fish on LBD for the aggregated data using ordinary linear regression.

### (c) Analyses of farm-level data

Analyses on aggregated scales may mask effects of predictor variables since averages over regions, or over years, are not necessarily representative for direct effects on farms. Therefore, we also performed more detailed analyses of farm-level data. We explored the relationship between monthly numbers of sea lice on 20 fishes and the explanatory variables: sea lice counts on the farm in earlier months, water temperature, mean fish weight, LBD, medical treatments; whether the farmed species was Atlantic salmon, the use of cleaner fish, and region. Water temperatures, sea lice counts, medical treatments and LBDs all tended to oscillate on an annual period (electronic supplementary material, figures S3–S4). To ensure that possible effects of temperature, LBD or medical treatment on sea lice counts were not merely owing to harmonized oscillations, a seasonal component comprising four sine and four cosine functions with periodicities of 12, 6, 4 and 3 months, respectively, were included in the model. Furthermore, to ensure that a trend in the sea lice count data, and possibly in explanatory variables, did not confound parameter estimates, a nonlinear overall trend modelled by five b-splines was included in the model [37].

In order to fit a model to the data, it is necessary to assume an appropriate probability distribution for the response variable, i.e. the number of sea lice per 20 fish. Count data are typically modelled assuming either a Poisson or negative binomial (NB) distribution [38]. The high proportion of zero counts in the data was not adequately captured by these distributions. We therefore used a two-component mixture model, which defines the response variable as a mixture of a NB and a Bernoulli distribution, termed a zero-inflated negative binomial (ZINB) distribution. The NB distribution was chosen because of overdispersion of the data in addition to the excess zeros (see electronic supplementary material, methods). The ZINB distribution allows zero counts to arise from two distinct mechanisms: either a count from a NB distribution (including the possibility of a zero count) or an excess zero count [39,40]. Covariates of each process may or may not be the same, affording flexibility to construct models with the potential to explain a higher degree of variability than assuming a single distribution. In the present analyses, we fitted the ZINB models using the function zinbinfl() from the pscl package (v. 1.02) in R [35], and compared models using the Akaike information criterion (AIC).

In initial analyses, we found that utilization of cleaner fish for controlling sea lice infection was significantly associated with high sea lice counts in the regression models. We do not anticipate high sea lice counts to be promoted by the use of cleaner fish. Since including the use of cleaner fish as an explanatory variable in the ZINB models does not contribute to gained insight into determinants of sea lice abundance, this variable was excluded from further farm-level analyses.

To investigate the robustness of our conclusions to potential problems in the data, separate ZINB models were run for each of the three regions and for subsets of data (see electronic supplementary material, table S4). (i) To investigate the potential impact of the use cleaner fish on parameter estimates, we fitted the model to the subset of data that included only salmonid cohorts with no report of cleaner fish use. (ii) Similarly, to investigate the potential impact of variation in salinity on parameter estimates, we fitted the model to the cohorts grown on the farms with less than median estimates for freshwater exposure. (iii) To investigate the potential impact of the change in reporting methodology in August 2009 on parameter estimates, we fitted the model to the data from before August 2009 only. Finally, (iv) to investigate the potential impact of correlations between salmonid cohorts within the same farm on parameter estimates and SE estimates, we fitted the model to the data from one random cohort per farm. Additional problems associated with temporal and spatial correlations were evaluated by estimating the temporal autocorrelation and the spatial variogram of the residuals.

## 3. Results

### (a) Exploratory analyses of region-level data

**Figure 2.**
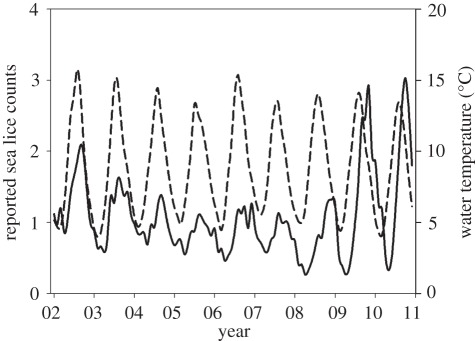
Sea lice abundance plotted as the mean of farm-reports of mobile stages of sea lice per fish for each month, and mean water temperature for each month in the period January 2002–December 2010. Solid line, mean count; dashed line, mean temperature.

**Figure 3.**
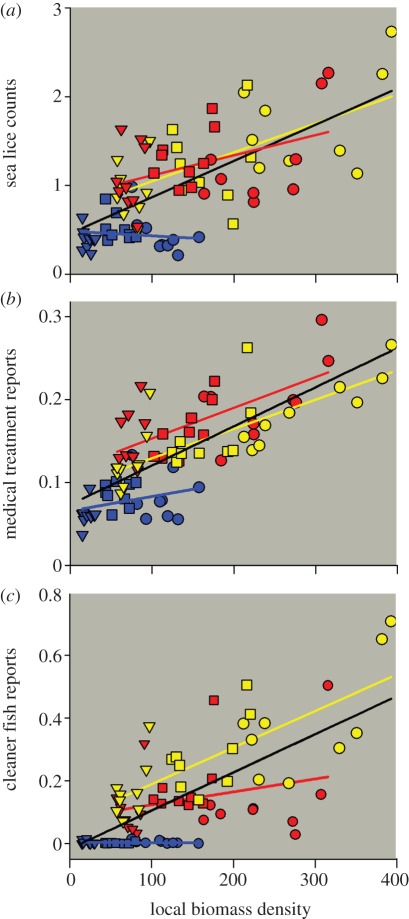
(*a*) Average counts of mobile sea lice per fish, (*b*) mean monthly proportion of farms treated medically against sea lice and (*c*) mean monthly proportion of farms reporting the use of cleaner fish, plotted against the average LBD of farmed salmonids. Estimates are given for each year (2002–2010), within years for farms located in areas with low (less than 33.3 percentile; triangles), medium (33.3–66.6 percentile; squares) and high (greater than 66.6 percentile; circles) LBD, and for each region (north, blue; mid, red; south, yellow). Lines are least-squares linear regressions through the data, where black represents all data and coloured lines represent corresponding regions.

**Figure 4.**
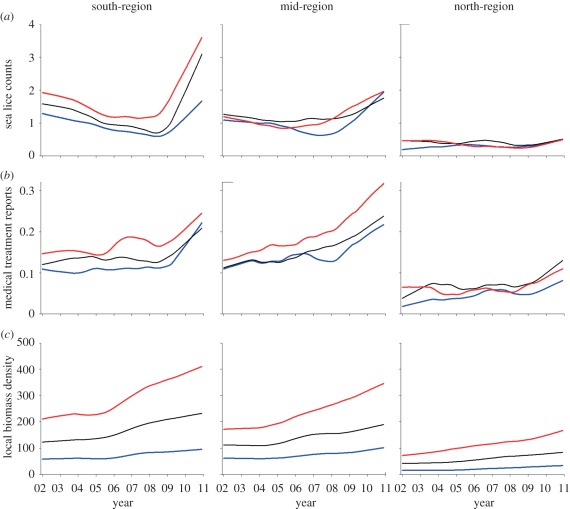
Locally weighted polynomial regression curves fitted to mean counts of (*a*) mobile stages of sea lice, (*b*) proportion of farms reporting medical treatments against sea lice and (*c*) mean local biomass of farmed salmonids (LBD) for the south-region, mid-region and north-region. For each month, the locally weighted polynomial regression curves are plotted separately for farms located in areas with low (less than 33.3 percentile), median (33.3–66.6 percentile) and high (greater than 66.6 percentile) LBD. All locally weighted polynomial regression curves were fitted using the lowess() function in R, with a smoother span of 0.4. Blue line, low LBD; black line, medium LBD; red line, high LBD.

Overall, the abundance of sea lice oscillated annually with a lag in relation to the annual fluctuations in water temperature, with low infection levels in March–April and peak infections in September–November (figure 2). To visually illustrate the main patterns in the relationship between the LBD of farmed salmon, sea lice abundances and intervention efforts, we display annual estimates of these variables for each coastal region (north-, mid- and south-regions; figure 1) and within regions for the terciles of farms with the lowest, intermediate and highest LBDs (figure 3). Overall, the average LBD was positively associated with the abundance of sea lice, medical treatments and the use of cleaner fish (electronic supplementary material, table S5). However, the patterns differed spatially. On this coarse scale, there was no evidence for LBD being associated with sea lice abundance, the use of medical treatments or cleaner fish in the north-region, but increasingly strong associations in the mid- and south-regions (electronic supplementary material, table S5). In addition, both sea lice abundance and intervention efforts were lower in the north-region than in the mid- and south-regions over the range of overlapping LBDs (figure 3), suggesting that lower water temperatures in the north reduced sea lice infection rates. Smoothed sea lice counts, medical treatments and LBDs over the study period are shown for the regions and LBD terciles in figure 4. Increasing sea lice counts and intensified medical treatments, especially in high LBD farms, are seen during the late part of the study period in the mid- and south-regions, but not in the north-region (figure 4).

### (b) Analyses of farm-level data

In our ZINB regression analyses of count reports of sea lice, the results from the two model parts in the ZINB regression model were consistent in their trends. Estimated positive effects in the NB model for the sea lice counts were, in general, accompanied by negative estimated effects on the probability of excess zeros (table 1). A strong temporal autocorrelation in sea lice counts on the salmonid farms suggested that counts in a given month depended on counts from the same farm in the previous four months. Furthermore, high water temperatures, high mean fish weights and the farmed species being Atlantic salmon rather than rainbow trout were all factors associated with high sea lice counts, while reported chemotherapeutic treatment in the previous month was associated with low sea lice counts. Importantly, high LBD was associated with high sea lice counts in the NB model and accompanied by low probabilities of excess zero reports in the logistic model (table 1). This result implies that high sea lice abundance was associated with areas of intense salmon farming in the farm-level data, as was the overall result from the analyses of region-level data.

**Table 1.** Parameter estimates, with standard errors in brackets, for the predictor variables entered into the zero-inflated negative binomial models to explain reported sea lice counts. Statistics for the total dataset and for separate models for the south-region, mid-region and north-region are shown (LBD, local biomass density; med. treat., medical treatment; temp, water temperature; bin, binary variables; *t*, month). Coefficients for the seasonal and time trends are not given in the table, but shown graphically in electronic supplementary material, figure S5.

	total dataset	south-region	mid-region	north-region
*negative binomial*				
intercept	1.52 (0.09)	1.65 (0.14)	1.61 (0.15)	0.27 (0.26)
north-region	−0.18 (0.02)			
south-region	0.075 (0.01)			
sea lice count (*t* − 1)	0.0081 (0.0002)	0.0067 (0.0002)	0.0081 (0.0003)	0.015 (0.0008)
sea lice count (*t* −2)	0.0029 (0.0002)	0.0029 (0.0002)	0.0026 (0.0003)	0.0043 (0.0007)
sea lice count (*t* −3)	0.0013 (0.0002)	0.0010 (0.0002)	0.0017 (0.0003)	0.0023 (0.0007)
sea lice count (*t* −4)	0.0011 (0.0002)	0.0011 (0.0002)	0.0012 (0.0003)	0.0015 (0.0008)
log(temp (°C) + 0.6; *t*)	0.60 (0.04)	0.56 (0.06)	0.55 (0.06)	1.10 (0.10)
log(fish weight (kg); *t*)	0.24 (0.01)	0.27 (0.01)	0.31 (0.01)	0.13 (0.02)
med. treat. (bin; *t* − 1)	−0.20 (0.02)	−0.24 (0.02)	−0.22 (0.03)	−0.21 (0.06)
LBD (*t* − 1)	0.14 (0.01)	0.15 (0.01)	0.12 (0.01)	0.11 (0.04)
Atlantic salmon	0.16 (0.03)	0.14 (0.03)	0.20 (0.05)	0.32 (0.09)
log(theta)	−0.39 (0.01)	−0.32 (0.01)	−0.22 (0.01)	−0.69 (0.02)
*zero inflation model*				
intercept	2.93 (0.22)	2.29 (0.34)	1.71 (0.41)	5.71 (0.48)
north-region	0.75 (0.05)			
south-region	0.52 (0.04)			
sea lice (bin; *t* − 1)	−2.55 (0.04)	−2.32 (0.05)	−2.21 (0.07)	−3.11 (0.12)
sea lice (bin; *t* − 2)	−0.72 (0.04)	−0.72 (0.06)	−0.80 (0.08)	−0.73 (0.10)
sea lice (bin; *t* − 3)	−0.41 (0.05)	−0.47 (0.06)	−0.48 (0.08)	−0.36 (0.11)
sea lice (bin; *t* − 4)	−0.30 (0.04)	−0.40 (0.06)	−0.07 (0.08)	−0.61 (0.10)
log(temp (°C) + 0.6; *t*)	−0.85 (0.09)	−0.28 (0.14)	−0.38 (0.17)	−2.43 (0.20)
log(fish weight (kg); *t*)	−0.13 (0.01)	−0.12 (0.02)	−0.18 (0.03)	−0.15 (0.03)
med. treat. (bin; *t* − 1)	0.32 (0.06)	0.27 (0.08)	0.31 (0.10)	0.58 (0.16)
LBD (*t* − 1)	−0.25 (0.02)	−0.21 (0.02)	−0.21 (0.04)	−0.27 (0.08)
Atlantic salmon	−0.62 (0.05)	−0.75 (0.06)	−0.24 (0.12)	0.11 (0.19)

After controlling for other predictor variables, including water temperature and LBD, in the ZINB regression model, there were additional effects of a seasonal trend and a time trend (electronic supplementary material, figure S5). Furthermore, there were still effects of region (table 1). Separate regression analyses for each of the three geographical regions suggested that fluctuations in water temperature had a stronger effect on sea lice counts in the north-region than in the south- and mid-regions (table 1). However, the effect of LBD was similar in all three regions even though there was no detected effect of LBD in the north in the analyses of region-level data (table 1 and figure 3). Finally, ZINB analyses of subsets of the data other than region were consistent with the analysis on the total dataset (electronic supplementary material, table S4).

In order to validate the full ZINB model, residuals were plotted against all explanatory variables and any remaining spatial correlation was explored by a variogram. No remaining patterns were observed. Furthermore, a mixed effects model of the residuals, with farm site as a random effect, was estimated. The standard deviation of the residuals and the random effect were 1.49 and 0.20, respectively. Hence, the random effect only accounted for 0.20^2^/1.49^2^ = 1.8% of the variance left in the residuals. A random effect with cohort of farmed salmonids nested within farms did not improve the fit. The residual first-order temporal autocorrelations were estimated for each cohort and found to be significant for less than 6 per cent of the cohorts. We conclude that no major systematic patterns were left in the residuals.

## 4. Discussion

LBD of farmed salmonids was associated with abundance of sea lice, such that high LBDs implied expectations of high sea lice counts. This association was consistent for different salmonid farming regions. Furthermore, high LBD was associated with intensified efforts to control sea lice infections. The positive LBD association with both sea lice abundance and control efforts accord with expectations of increased production of sea lice transmission stages at elevated host densities of farmed salmonids, and suggests that local host density is a main factor determining the infection pressure experienced by farmed salmonids in Norway. Given the prevailing production system for farming salmonids, parasitic sea lice may accordingly limit local densities of farmed salmonids since efforts to control infections are likely to surpass economically or environmentally sustainable levels at some host density. Hence, we conclude that sea lice represents a potent density-dependent negative feedback mechanism that may limit growth in salmonid farming in Norway.

Over the late part of the study period, peak abundances of sea lice and frequencies of medical treatments increased, especially in intensive farming areas. This pattern may suggest that chemical control of sea lice infections became less feasible in these areas, possibly owing to evolving resistance in the sea lice population towards commonly used drugs. The Norwegian Food Safety Authority reports increasing incidences of reduced sensitivity and/or resistance to medical treatments, as well as changes in the composition of the active substances used in chemotherapy and increasing quantities of drugs applied to farmed salmon to control sea lice [41]. Reduced sensitivity and/or resistance in sea lice to a range of different medical substances, and in different geographical areas, have been documented [42–46]. The efficacy of treatments has also been shown to decrease over time [32], and suggested to depend on the frequency of treatments by a given drug [31]. Given evolving resistance to treatment in sea lice and that this is reinforced by increasing densities of farmed salmon owing to increasing frequencies of treatment, a worst case scenario will be that resistant sea lice spread from high LBD areas and reduces sustainable levels of salmon farming on extended spatial scales along the coast. Alternatively, new methods to control sea lice infections may appear. There is focus on moving production from open to closed systems. Research and development activities are also directed at developing new drugs or combinations of drugs for medical treatments, developing vaccines and farming of cleaner fish [47,48], all testifying to the importance of the problem when using the production technology applied today.

As expected, sea lice counts were influenced by water temperatures [25]. The north–south gradient in temperature, in addition to generally low densities of farmed salmonids in the north, probably explains low sea lice counts in the north-region. Also, intervention efforts were low in the north-region, compared with the south- and mid-regions, over the range of overlapping LBDs. The temperature effect suggests that colder water temperature in the north reduces sea lice transmission. There is, therefore, a reason to believe that comparable levels of LBD will entertain smaller sea lice populations in the north than in the south, all conditions apart from temperature being equal.

In our initial analyses, we found that the utilization of cleaner fish for controlling sea lice infection was significantly associated with high sea lice counts (see §2). The reason for this finding is likely to be that efforts to control infections are elevated when farms experience high sea lice abundances. Such a positive association could potentially also have been attained for medical treatments. The difference between the two intervention variables in their association with sea lice abundance is probably caused by a subtle long-term effect of cleaner fish, whereas the effect of medical treatments are more instant and stronger. Nevertheless, our finding emphasizes the need for experimental studies to evaluate control effects of cleaner fish and other interventions. Also other variables that are expected to affect sea lice population dynamics need further study, e.g. salinity [34,49] and the possible development of resistance/loss of sensitivity of sea lice to medical treatments [31,32]. These examples, and the potential for confounding between predictor variables, emphasize that caution should be taken when evaluating the strength of effects in the present analyses. The present large dataset, however, allows for analyses of subsets of data to check the consistency of main effects across possible confounders. In the electronic supplementary material, table S4, we report from analyses of different subsets of the data where we exclude all records where cleaner fish were used; exclude 50 per cent of the farm locations estimated to be most strongly exposed to freshwater; and include data only up to August 2009 when counting procedures changed. In all these analyses, we have a consistent positive effect of LBD on the abundance of sea lice infection. This suggests that the conclusion that abundance of sea lice infection depends on LBD is robust, in that it does not depend on certain parts of the data or that potential confounding variables have not been included in the models.

Norwegian regulations dictate an upper threshold to the mean number of sea lice per farmed salmonid in a farm, to reduce harmful effects of sea lice on both wild and farmed salmonids [50]. This threshold does not account for spatial or temporal heterogeneity in host densities, which we find to be a main determinant of sea lice abundance. With a continued increase in the density of farmed salmon, our analyses suggest that the current management regime will lead to increasing sea lice infection pressure in fish farms, as well as increasing efforts of chemotherapeutic control and hence the risk of development and the spread of treatment resistance [31,32]. To counter this development, we believe regulations will need to go from a threshold defined for the average infection per fish to a threshold based on a measure of the spatial sea lice density.

The rapid development of a highly industrialized production of farmed salmon has contributed to a strong belief in continued growth in aquaculture [7,9]. We propose that infectious diseases represent a potent density-dependent negative feedback mechanism that may limit such growth.
